# *Dactylogyrus* spp. (Dactylogyridae, Monogenea) from tinfoil barb, *Barbonymus schwanenfeldii* imported into South Africa: morphometric and molecular characterisation[Fn FN1]

**DOI:** 10.1051/parasite/2023031

**Published:** 2023-08-11

**Authors:** Prince S. Molokomme, Michal Benovics, Wilmien J. Luus-Powell, Linda P. Lukhele, Iva Přikrylová

**Affiliations:** 1 DSI-NRF SARChI Chair (Ecosystem Health), Department of Biodiversity, University of Limpopo Sovenga 0727 South Africa; 2 Department of Botany and Zoology, Faculty of Science, Masaryk University 611 37 Brno Czech Republic; 3 Department of Zoology, Faculty of Sciences, Comenius University in Bratislava 841 04 Bratislava Slovakia; 4 Water Research Group, Unit for Environmental Sciences and Development, North-West University Potchefstroom 2520 South Africa

**Keywords:** Monogenea, *Dactylogyrus*, *Barbonymus*, Ornamental fish, South Africa

## Abstract

This study reports on three species of *Dactylogyrus* Diesing, 1850 (Dactylogyridae) collected from tinfoil barb, *Barbonymus schwanenfeldii* (Bleeker) which were imported into South Africa as ornamental fish from Sri Lanka and Thailand. Supplementary morphometric characterisation and molecular data (partial 18S and 28S rDNA, and ITS1 region sequences) are presented for *Dactylogyrus lampam* (Lim & Furtado, 1986), *Dactylogyrus tapienensis* Chinabut & Lim, 1993 and *Dactylogyrus viticulus* Chinabut & Lim, 1993. Prevalence of *Dactylogyrus* spp. infection was 87% and 80% for fish from Sri Lanka and Thailand, respectively. Composition of the parasites between the fish of each origin differed. All three species were found to infect fish from Thailand, but only *D. lampam* was present on the fish received from Sri Lanka. Phylogenetic analysis revealed the position of studied species, with *D. lampam* clustering within the lineages of varicorhini-type species, while *D. tapienensis* and *D. viticulus* form a sister lineage to *Dactylogyrus* spp. associated with *Cyprinus carpio* L. and *Carassius* spp., species parasitising central African large cyprinids (*Labeo* Cuvier), and species parasitising African and Middle Eastern *Carasobarbus* spp.

## Abstract (fr)

Cette étude porte sur trois espèces de *Dactylogyrus* Diesing, 1850 (Dactylogyridae), prélevées sur des *Barbonymus schwanenfeldii* (Bleeker) qui ont été importés en Afrique du Sud comme poissons d’ornement depuis le Sri Lanka et la Thaïlande. Une caractérisation morphométrique et des données moléculaires supplémentaires (ADNr 18S et 28S partiels et séquences de la région ITS1) sont présentées pour *Dactylogyrus lampam* (Lim & Furtado, 1986), *Dactylogyrus tapienensis* Chinabut & Lim, 1993 et *Dactylogyrus viticulus* Chinabut & Lim, 1993. La prévalence de l’infection par les *Dactylogyrus* spp. était respectivement de 87 % et 80 % pour les poissons du Sri Lanka et de Thaïlande. La composition des parasites entre les poissons des deux origines différait. Les trois espèces infectaient les poissons de Thaïlande, mais seul *D. lampam* était présent sur les poissons du Sri Lanka. L’analyse phylogénétique a révélé la position des espèces étudiées, *D. lampam* se regroupant dans les lignées d’espèces de type varicorhini, tandis que *D. tapienensis* et *D. viticulus* forment une lignée sœur des *Dactylogyrus* spp. associés à *Cyprinus carpio* L. et *Carassius* spp., espèces parasitant les grands cyprinidés d’Afrique centrale (*Labeo* Cuvier), et espèces parasitant les *Carasobarbus* spp. d’Afrique et du Moyen-Orient.

## Introduction

Southeast Asia is home to one of the world’s greatest diversities of freshwater fish. The Cyprinoidei is the most diverse taxon and cyprinoids dominate nearly every water body in the area [70]. In the region, the most speciose fishes are those of Cyprinidae, namely *Barbodes* Bleeker, *Barbonymus* Kottelat, *Cyclocheilichthys* Bleeker, *Hampala* Kuhl & van Hasselt, *Osteochilus* Gunther*, Puntius* Hamilton, and *Tor* Gray [16]. Tinfoil barb, *Barbonymus schwanenfeldii* (Bleeker), is one of five valid species of the *Barbonymus*, all native to Southeast Asia [17]. *Barbonymus schwanenfeldii* is a tropical river fish that is abundant in Peninsular Malaysia’s rivers and lakes [26].

Species richness and distribution of parasites in host species are usually closely related to the history, dispersion, and diversity of their hosts [3]. *Dactylogyrus* Diesing, 1850 (Monogenea) is known for its high species richness, with over 900 nominal species [19] that are primarily restricted to species of the Cyprinoidei [57]. In the Southeast Asia region, *Dactylogyrus* spp. are known on three out of five currently known *Barbonymus* spp., namely *B. altus* (Gunther), *B. gonionotus* (Bleeker) and *B. schwanenfeldii* (Bleeker) [11]. There are seven species of *Dactylogyrus* reported from these hosts, e.g., *D. kanchanaburiensis* Chinabut & Lim, 1993, *D. lampam* (Lim & Furtado, 1986)*, D. pseudosphyrna* Chinabut & Lim, 1993, *D. sianensis* Chinabut & Lim, 1993*, D. tapienensis* Chinabut & Lim, 1993*, D. tonguthaii* Chinabut & Lim, 1993, and *D. viticulus* Chinabut & Lim, 1993, with all from *B. gonionotus* and with four and three *Dactylogyrus* spp. recorded from *B. schwanenfeldii* and *B. altus*, respectively [11]. Moreover, one of these species, *D. pseudosphyrna*, can also be found in Thailand on a non-*Barbonymus* host, *Cyclocheilichthys enoplos* (Bleeker) [11].

For a long time, most taxonomic studies on *Dactylogyrus* spp. have been based on morphometry of the attachment organ’s sclerites and hard parts of reproductive organs only (i.e., male copulatory organ and vagina) [36, 39, 42]. However, recently it has been emphasised and demonstrated that the ideal way forward to secure accurate identification is the integrated approach combining morphometric and molecular data [1, 6, 40, 44, 45]. Currently, there are 898 entries available in the GenBank database for a variety of *Dactylogyrus* spp. (search December 2022) with most items found for *Dactylogyrus vistulae* Prost, 1957. Out of seven *Dactylogyrus* species that can be found on *Barbonymus* hosts, only a partial 18S and ITS1 rDNA region sequence for *D. lampam* is available in the database, representing only a direct entry from Malaysia and not linked to published results. There is, in general, a substantial lack of genetic data for *Dactylogyrus* spp. from Asia compared to the species from Europe or North America which have recently been studied intensively [3–8, 48, 51, 53].

Aquaristics is a popular hobby worldwide and in connection with this, ornamental trade has become a well-functioning industry with more than 50% of recognised countries being involved [68]. The translocation of millions of ornamental fish every year poses a risk of introduction of non-native parasites together with their hosts. It has been documented that fish were responsible for spreading their parasites into non-native regions more than other animals [30]. Several studies have confirmed the presence of monogeneans in imported or introduced fish [35, 65], with cases of spill-over of introduced parasites to native fish [25].

This study originally aimed to screen ornamental fish *B*. *schwanenfeldii* for the presence of parasites that may have been imported into South Africa. The finding of three *Dactylogyrus* species provided the opportunity to produce missing molecular data for the species, as well as supplement original morphometric descriptions. Additionally, the phylogenetic relationship to the other species of the genus could be determined.

## Materials and methods

### Parasite sampling

A total of 44 specimens of tinfoil barb, *B. schwanenfeldii* (TL = 7.3–10.8 cm; mean 8.80 ± 1.04) originating in Thailand and Sri Lanka were imported into South Africa through a well-established importing company. All samples were acclimatised upon arrival, following the protocol provided by the importing company and placed in 50-litre glass aquaria with a continuous oxygen supply generated from portable aerators and water heated to a temperature of 24 °C. Each fish was killed following the protocol for the Ethical Handling of Ectothermic Vertebrates by percussive stunning and cervical transection (University of Limpopo Animal Research and Ethics Committee Clearance AREC/05/22: PG). Gills of freshly killed fish specimens were extracted, placed in a Petri dish containing distilled water, and examined for the presence of parasites using a stereomicroscope Leica EZ4 (Leica Microsystems GmbH, Wetzlar, Germany). Internal organs were also screened for the presence of parasites. Total parasite count on the gills was noted and only a representative sample was preserved. Monogeneans were removed from the gills using fine needles and prepared as in Řehulková et al. [43]. Specimens used for morphological examination were completely flattened under coverslip pressure in order to best expose their sclerotised structures (haptoral and reproductive sclerites) and fixed in a mixture of ammonium picrate-glycerin [31]. Specimens used for DNA analysis were bisected using fine needles. Subsequently, one-half of the body (either the posterior part haptoral sclerites or the anterior part containing the male copulatory organ) was fixed in 96% ethanol for later DNA extraction. The mounted specimens were studied using a phase-contrast microscope Olympus BX51 (Olympus, Tokyo, Japan) equipped with a camera and imaging software (Stream Essential, Soft Imaging System GmbH 1986 version 1.5.1, Olympus). Drawings of the sclerotised structures were made with the aid of a camera lucida and digitised with Adobe Illustrator^®^ software (Adobe Inc., San Jose, CA, USA) and a Wacom Intuos Pro drawing tablet (Wacom, Saitama, Japan), following Truter et al. [66]. Measurements were taken using a phase-contrast microscope Olympus BX51 following the scheme presented in Řehulková et al. [44]. All measurements (in micrometres) are provided as the range followed by the mean and number of measured specimens in brackets. The numbering of hook pairs (in Roman numerals I–VII) is that suggested by Mizelle [33]. Epidemiological characteristics such as parasite prevalence, *P* (percentage of infected hosts), the intensity of infection, IF (minimum and maximum number of parasites per infected host) and mean intensity of infection, MI (the mean number of parasites per infected host) were calculated for the cumulative numbers of *Dactylogyrus* spp. according to Bush et al. [9]. Voucher specimens collected during the present study and transferred into Canada balsam [15] were deposited at the Institute of Parasitology of the Czech Academy of Sciences (IPCAS), České Budějovice, Czech Republic, and in the National Museum, Bloemfontein (NMB), South Africa. A paratype specimen *D. lampam* (IPCAS M-286) was studied for comparative purposes.

### DNA extraction and PCR amplification

Prior to DNA analysis, monogeneans were identified based on morphology and then preserved in 96% ethanol. Each *Dactylogyrus* part preserved in ethanol was dried using a vacuum centrifuge. DNA was extracted using the standard protocol (DNeasy Blood & Tissue Kit, Qiagen, Hilden, Germany). The partial 18S, entire ITS1, and partial 5.8S regions were amplified using the reverse primer S1 (5′ – ATTCCGATAACGAACGAGACT – 3′) and reverse primer IR8 (5′ – GCTAGCTGCGTTCTTCATCGA – 3′), which anneal to the segments of DNA coding 18S and 5.8S, respectively [47]. Amplification reactions followed protocols optimised in Benovics et al. [5]. The partial 28S region was amplified using the forward primer C1 (5′ – ACCCGCTGAATTTAAGCA – 3′) and reverse primer D2 (5′ – TGGTCCGTGTTTCAAGAC – 3′) [24], following the PCR protocol optimised by Šimková et al. [49]. The PCR products (~1000 bp for 18S, ITS1, and 5.8S, and ~ 800 bp for partial 28S) were checked on 1% agarose gel and purified using an ExoSAP-IT kit (Ecoli, Bratislava, Slovakia), following the standard protocol. For sequencing, the commercial services of Macrogen Europe (Amsterdam, Netherlands) were employed, and sequencing was carried out using the same primers as for the amplification reaction.

### Phylogenetic analyses

In order to assess the molecular phylogenetic relationships of the three collected *Dactylogyrus* spp., ortholog sequences of selected *Dactylogyrus* spp. parasitising cyprinid fish hosts (family Cyprinidae according to the recent revision by Tan and Armbruster [59]) in Africa, Asia, and Europe, and one outgroup taxon *Ancyrocephalus percae* (Ergens, 1966) (selected as phylogenetically proximal taxon) according to Mendoza-Palmero et al. [32] were retrieved from GenBank (full list of species given in Table 1). Previous phylogenetic studies confirmed unique phylogenetic associations among *Dactylogyrus* of cyprinids and linked their diversification with the historical speciation of respective hosts (e.g., [1, 7, 52]). These studies also recorded the congruency between the molecular and morphological phylogenies in the *Dactylogyrus* of Cyprinidae. The sequences were aligned using the Fast Fourier transform algorithm in MAFFT [27] using the G-INS-I refinement method, and the ends were manually trimmed to unify their length. All parameters for phylogenetic analyses were treated as variables, therefore GTR (the general time-reversible evolutionary model) was selected as the preferred evolutionary model. The shape parameter of the gamma distribution (*G*) and the proportion of invariable sites (*I*) were selected using jModelTest v 2.1.10 [13, 20]. Phylogenetic analyses using maximum likelihood (ML) were computed employing RAxML v 8.1.12 [55, 56]. The best ML tree was selected from 100 iterations, and support for the branching pattern was validated through 10^3^ pseudoreplicates. Phylogenetic analyses of Bayesian inference (BI) were carried out in MrBayes v 3.2 [46], and the resulting tree was constructed using the Metropolis-coupled Markov chain Monte Carlo algorithm. Four concurrent chains (one cold and three heated) ran for 5 × 10^6^ generations, sampling trees every 100 generations. The first 30% of trees were discarded as a relative burn-in period after checking that the standard deviation split frequency fell below 0.01. Results were checked in Tracer v 1.7.1 [41] to assess convergence. Posterior probabilities were calculated as the frequency of samples recovering particular clades.

**Table 1:** List of *Dactylogyrus* spp., their host species, country of collection, and GenBank accession number for 28S sequences used for phylogenetic reconstruction. Newly generated sequences are given in bold.

Dactylogyrus species	Host species	Country of collection	Accession number
*Ancyrocephalus percae*	*Perca fluviatilis*	Germany	KF499080
*Dactylogyrus achmerowi*	*Cyprinus carpio*	Iran	MF979966
*Dactylogyrus affinis*	*Barbus cyri*	Iran	MZ031054
*Dactylogyrus anchoratus*	*Carassius gibelio*	Croatia	KY863555
*Dactylogyrus andalousiensis*	*Luciobarbus comizo*	Spain	MN338207
*Dactylogyrus atlasensis*	*Luciobarbus pallaryi*	Morocco	KY629356
*Dactylogyrus balistae*	*Luciobarbus bocageii*	Portugal	MN338205
*Dactylogyrus balkanicus*	*Barbus tyberinus*	Italy	MN973809
*Dactylogyrus barbuli*	*Luciobarbus xanthopterus*	Iraq	MZ031063
*Dactylogyrus benhoussai*	*Luciobarbus yahyahouii*	Morocco	MN973815
*Dactylogyrus bocageii*	*Luciobarbus bocageii*	Portugal	KY629347
*Dactylogyrus borjensis*	*Luciobarbus yahyahouii*	Morocco	MN973819
*Dactylogyrus brevicirrus*	*Labeo parvus*	Senegal	KY629362
*Dactylogyrus carassobarbi*	*Carassobarbus luteus*	Iraq	MZ031060
*Dactylogyrus carpathicus*	*Barbus tyberinus*	Italy	MN973810
*Dactylogyrus crivellius*	*Barbus tyberinus*	Italy	MK434949
*Dactylogyrus doadrioi*	*Luciobarbus guiraonis*	Spain	KY629346
*Dactylogyrus draaensis*	*Luciobarbus lepineyi*	Morocco	MN973816
*Dactylogyrus dyki*	*Barbus balcanicus*	Greece	MG792970
*Dactylogyrus extensus*	*Cyprinus carpio*	China	AY553629
*Dactylogyrus falciformis*	*Cyprinus carpio*	Czech Republic	MZ031061
*Dactylogyrus falcilocus*	*Labeo coubie*	Senegal	KY629365
*Dactylogyrus falsiphallus*	*Luciobarbus maghrebensis*	Morocco	KX578024
*Dactylogyrus fimbriphallus*	*Luciobarbus lepineyi*	Morocco	KY629357
*Dactylogyrus formosus*	*Carassius gibelio*	Croatia	MG792984
*Dactylogyrus goktschaicus*	*Barbus cyri*	Iran	MZ031055
*Dactylogyrus gracilis*	*Capoeta buhsei*	Iran	MZ031056
*Dactylogyrus guadianensis*	*Luciobarbus comizo*	Spain	MN338209
*Dactylogyrus inexpectatus*	*Carassius auratus*	Czech Republic	AJ969945
*Dactylogyrus ksibii*	*Luciobarbus ksibii*	Morocco	MN973811
*Dactylogyrus kulindri*	*Carassobarbus fritschii*	Morocco	KY629354
*Dactylogyrus kulwieci*	*Luciobarbus xanthopterus*	Iraq	MZ031064
*Dactylogyrus labei*	*Catla catla*	India	JX566720
***Dactylogyrus lampam***	*Barbonymus schwanenfeldii*	Thailand	OR077123
*Dactylogyrus legionensis*	*Luciobarbus graellsi*	Spain	MN338210
*Dactylogyrus lenkorani*	*Capoeta buhsei*	Iran	MZ031057
*Dactylogyrus lenkoranoides*	*Barbus haasi*	Spain	MN338202
*Dactylogyrus leonis*	*Labeo coubie*	Senegal	KY629360
*Dactylogyrus linstowi*	*Luciobarbus capito*	Iran	MZ031062
*Dactylogyrus malleus*	*Barbus barbus*	Czech Republic	KY201112
*Dactylogyrus marocanus*	*Carassobarbus fritschii*	Morocco	KY629355
*Dactylogyrus mascomai*	*Luciobarbus bocageii*	Spain	MN338206
*Dactylogyrus matlopong*	*Labeobarbus aenus*	South Africa	ON391043
*Dactylogyrus oligospirophallus*	*Labeo coubie*	Senegal	KY629361
*Dactylogyrus omenti*	*Aulopyge huegelii*	Bosnia and Herzegovina	KY201105
*Dactylogyrus petenyi*	*Barbus balcanicus*	Greece	KY201113
*Dactylogyrus prespensis*	*Barbus prespensis*	Greece	KY201110
*Dactylogyrus pulcher*	*Capoeta razii*	Iran	MZ031058
*Dactylogyrus quangfami*	*Cirrhinus molitorella*	China	EF100536
*Dactylogyrus remi*	*Luciobarbus graecus*	Greece	KY201115
*Dactylogyrus romuli*	*Luaciobarbus albanicus*	Greece	KY201114
*Dactylogyrus scorpius*	*Luciobarbus rifensis*	Morocco	KX553860
*Dactylogyrus senegalensis*	*Labeo senegalensis*	Senegal	KY629363
*Dactylogyrus* sp.	*Sikukia flavicaudata*	China	MH790264
***Dactylogyrus tapienensis***	*Barbonymus schwanenfeldii*	Thailand	OR077124
*Dactylogyrus titus*	*Labeo senegalensis*	Senegal	KY629364
*Dactylogyrus varius*	*Luciobarbus massaensis*	Morocco	MN973814
*Dactylogyrus vastator*	*Carassius gibelio*	Croatia	MZ031059
***Dactylogyrus viticulus***	*Barbonymus schwanenfeldii*	Thailand	OR077125
*Dactylogyrus volutus*	*Carassobarbus fritschii*	Morocco	KY629353
*Dactylogyrus zatensis*	*Carassobarbus fritschii*	Morocco	KY629352

## Results

Specimens of *Dactylogyrus* spp., were found on the gills of *B*. *schwanenfeldii* received from Sri Lanka (*n* = 24, *P* = 87%, IF = 7–98, MI = 44.3) and Thailand (*n* = 20, *P* = 80%, IF = 1–54, MI = 21.7). The morphometric evaluation confirmed the presence of three species of *Dactylogyrus*, *D. lampam*, *D. tapienensis* and *D. viticulus* from the host specimens received from Thailand, while those from Sri Lanka were infected by *D. lampam* only. New 28S and 18S + ITS1 rDNA sequences were obtained from *D. tapienensis* and *D. viticulus,* only a 28S rDNA sequence was successfully obtained for *D. lampam,* and their phylogenetic relationship within the genus was inferred. Detailed redescriptions based on both morphometric and molecular data are presented below in alphabetic order. No other parasites were found on or in the studied specimens.

Order Dactylogyridea Bychowsky, 1937

Family Dactylogyridae Bychowsky, 1933

### *Dactylogyrus lampam* (Lim & Furtado, 1986) (Fig. 1)

Type-host: *Barbonymus schwanenfeldii* (Bleeker, 1853).

**Figure 1:**
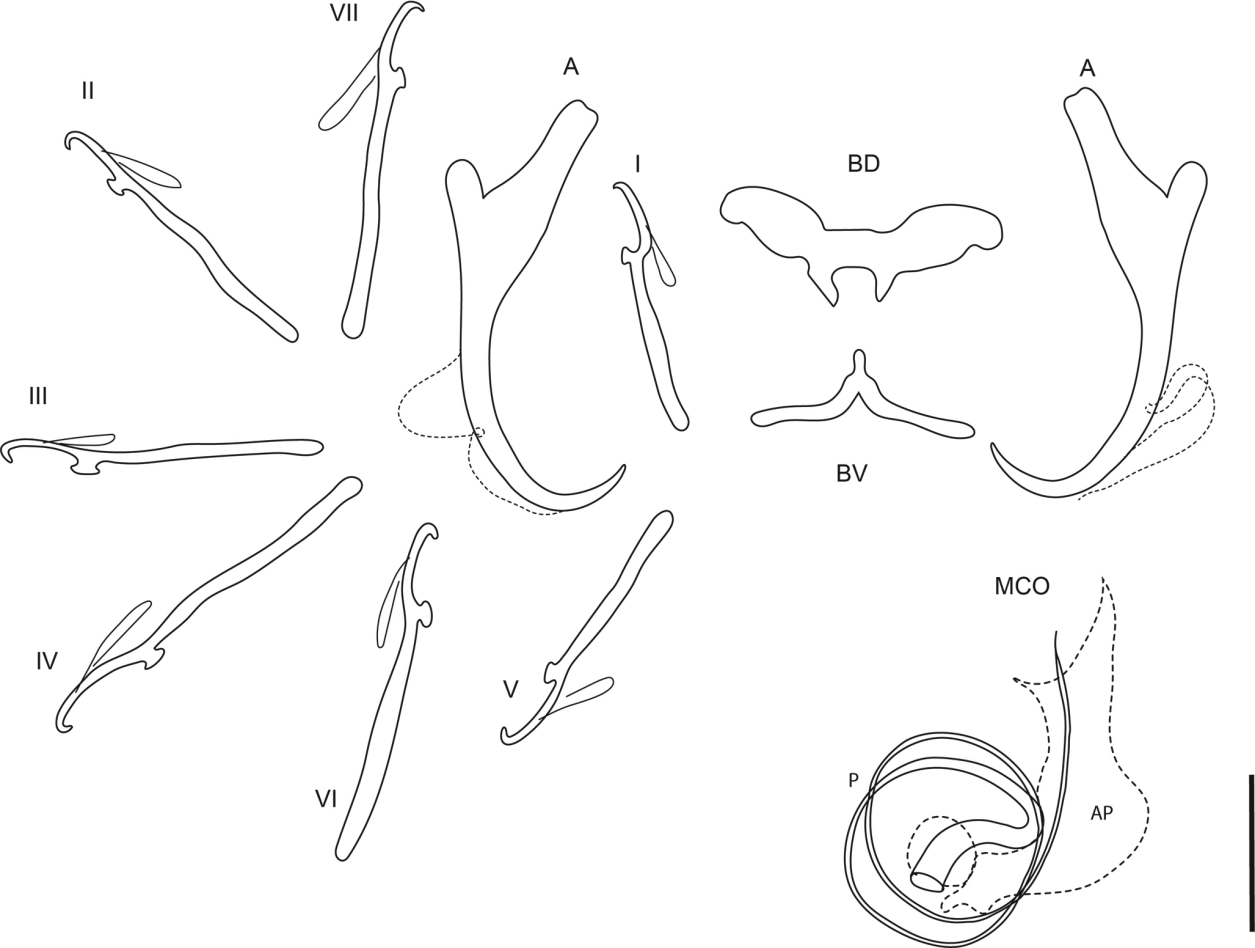
Line drawings of sclerotised structures of *Dactylogyrus lampam* (Lim & Furtado, 1986) ex *Barbonymus schwanenfeldii.* A, anchor; BD, dorsal bar; BV, ventral bar; I–VII, hooks; MCO, male copulatory organ; AP, accessory piece; P, penis. Scale bar 10 μm.

Other host: *Barbonymus gonionotus* (Bleeker, 1850).

Type-locality: Bukit Merah Reservoir, Merah, Malaysia.

Present records: Sri Lanka, Thailand.

Infection site: Gills.

Material deposited: 2 voucher specimens M-776 and 2 voucher specimens NMB P 948-9.

DNA sequence: A nucleotide sequence of partial 28S rDNA (823 bp; access. No. OR077123).

Redescription [based on 15 adult flattened specimens in GAP.] Composition of body as per definition by Gussev [21]. Body 222–325 (281; *n* = 9) long, greatest width 51–71 (67; *n* = 9) usually between 1/3 and mid length. Haptor differentiated from body proper, 33–55 (46; *n* = 9) long, 55–77 (66; *n* = 9). Measurements of haptoral sclerites and MCO are given in Table 2. One pair of anchors of varicorhini-type [21], with well develop inner and outer roots. Outer root short with rounded base, inner root elongated, about 1/3 of anchor shaft length. Shaft narrows in inner side before turning in point. Transversal bars of varicorhini-type: dorsal bar shape as birds-like wings, narrowing towards rounded ends. Thin V-shaped ventral bar with well-developed short middle process. Hooks seven pairs, all of similar shape, very fine point, robust shank without narrowing. Uneven in size, pairs I and V slightly smaller. MCO spiral shaped, composed of a 2–2.5× coiled tube with a slightly sclerotised accessory piece. Vagina not observed.

**Table 2:** Measurements of three *Dactylogyrus* spp. ex *Barbonymus schwanenfeldii* from the present study compared with values given in the original species descriptions. Min–max, (mean, number of measurements).

Species	*Dactylogyrus lampam*		*Dactylogyrus tapienensis*		*Dactylogyrus viticulus*	
	Lim and Furtado, 1986	Present study	Chinabut and Lim 1993	Present study	Chinabut and Lim 1993	Present study
Country	Malaysia	Thailand, Sri Lanka	Thailand	Thailand	Thailand	Thailand
ATL	30–34 (32)	26–30.3 (27.3; 14)	46–62 (59)	52.5–64.8 (58.3; 15)	57–65 (60)	55.2–67 (61.1; 15)
ASL	25–28 (27)	20–25.2 (21.9; 14)	38–46 (42)	38.8–49.3 (42.6; 15)	40–43 (41)	38.8–48.5 (44; 15)
APL	8–10 (8)	6–8.5 (7.4; 14)	12–19 (17)	16.3–20.1 (18.4; 15)	20–24 (20)	19.5–25.5 (21.7; 15)
AIRL	8–10 (8)	7.5–8.9 (8.3; 14)	14–26 (21)	16.5–22.5 (20; 15)	22–25 (23)	18.8–25.3 (22; 15)
AORL	1–2 (2)	1.7–3.2 (2.4; 14)	4–10 (7)	4.7–7.5 (6.2; 15)	4–7 (6)	3.1–5.6 (4.4; 15)
DBW	24–27 (25)	18.2–22.4 (19.7; 11)	24–26 (25)	20–24.4 (22.5; 15)	16–20 (18)	15.2–20.7 (17.5; 15)
VBW	22–25 (23)	16.7–19.9 (18.3; 14)	–	–	–	–
VBWL	–	8.5–11.5 (9.6; 14)	–	–	–	–
VBMPL	–	2–4.1 (2.8; 14)	–	–	–	–
LMCO	19–22 (21)	14.5–18.3 (16.5; 14)	64–90 (86)	83–93.4 (89; 15)	42–46 (44)	43.5–50.7 (45; 15)
HL	16–26		25 (23–28)		32–38 (35)	
I		15.4–17.8 (16.7; 12)		16.3–18.6 (17.5; 13)		26.5–31.5 (28.2; 12)
II		18.3–21.1 (19.3; 12)		18.9–21.2 (20.1; 13)		26.3–34.4 (30.1; 12)
III		19.8–22.5 (21.5; 12)		21–22.6 (21.7; 12)		28.5–35.9 (30.9; 13)
IV		22.3–26.2 (24.5; 12)		22.1–25 (23.3; 13)		29.3–35.4 (31.4; 13)
V		15.6–17.8 (17.1; 12)		16.8–18.3 (17.7; 9)		26.2–33.8 (28.5; 11)
VI		20.1–22.6 (21.4; 12)		20.8–22.9 (21.9; 13)		27.9–34.5 (29.8; 13)
VII		20–23 (21.8; 12)		21–23.3 (22.1; 13)		28.5–34.6 (30.9; 13)

ATL – anchor total length, ASL – anchor shaft length, APL – anchor point length, AIRL – anchor inner root length, AOLR – anchor outer root length, DBW – dorsal bar width, VBW – ventral bar width, VBWL – ventral bar wing length, VBMPL – ventral bar middle process length, LMCO – length of male copulatory organ, HL – hooklets length.

Remarks: The species was originally described by Lim and Furtado [28] as *Dactylogyrus puntii* in Malaysia, but later renamed on *D. lampam* by Lim (1991) as there was already an existing species of that name, *Dactylogyrus puntii* Buschkiel, 1930 described from *Barbodes lateristriga* (Valenciennes), formerly *Puntius lateristriga*, from Java. The morphology of the specimens collected during the present study corresponds with drawings presented by Lim and Furtado [28], but in size of haptoral hard parts newly collected specimens are slightly smaller. In the original species description of their species, Lim and Furtado [28] mention a similar species, *Dactylogyrus quangfami* Ha Ky, 1971, parasitic on *Cirrhinus molitorella* (Valenciennes) in Vietnam. From *D. quangfami*, *D. lampam* differs in (1) the general morphology of anchors – *D. quangfami* has a sturdier body of the shaft compared to *D. lampam*, (2) the morphology of the hooks – no evident narrowing of the shank in *D. lampam* vs. 1/3 thinner part of shank after the sickle proper of *D. quangfami*, and (3) the shape of a ventral bar – shown in Ha Ky [22] for *D. quangfami* as a simple fine type while *D. lampam* has a V-shape ventral bar with the middle process. From other *Dactylogyrus* spp. with the presence of two bars and those described from small cyprinids in Asia, *D. lampam* is similar to *D. fasciculi* Lim & Furtado, 1986, *D. binotati* Lim & Furtado, 1986, *D. perakensis* Lim & Furtado, 1986 and *D. kanchanburiensis*, in the general morphology of anchors, but none of *D. fasciculi*, *D. binotati*, *D. perakensis* or *D. kanchanaburiensis* do have a ventral bar with the pronounced middle process. Moreover, *D. kanchanaburiensis* has larger anchors 30–53 μm (48, inner/total length) than those of *D. lampam* (26–30 μm; 27, present study).

### *Dactylogyrus tapienensis* Chinabut & Lim, 1993 (Fig. 2)

Type-host: *Barbonymus gonionotus* (Bleeker, 1850).

**Figure 2:**
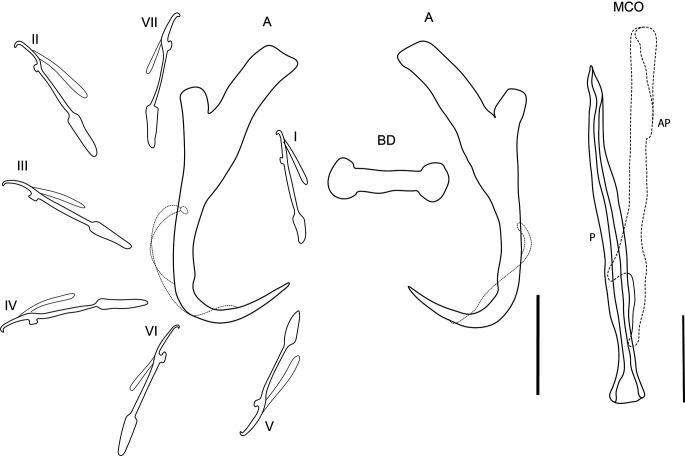
Line drawings of sclerotised structures of *Dactylogyrus tapienensis* Chinabut & Lim, 1993 ex *Barbonymus schwanenfeldii.* A, anchor; BD, dorsal bar; I–VII, hooks; MCO, male copulatory organ; AP, accessory piece; P, penis. Scale bar 20 μm.

Other Hosts: *Barbonymus altus* (Gunther, 1868), *Barbonymus schwanenfeldii* (Bleeker, 1853).

Type locality: Vachiralongkorn Reservoir, Kanchanaburi Province, Thailand.

Present record: Thailand.

Infection site: Gills.

Material deposited: 2 voucher specimens M-777 and 2 voucher specimens NMB P 950-1.

DNA sequence: A nucleotide sequence of partial 28S rDNA (845 bp; access. No. OR077124) and nucleotide sequences representing a fragment (975 bp; access. No. OR081826) including partial 18S rDNA (487 bp), and the ITS1 region (488 bp).

Redescription [based on 15 adult flattened specimens in GAP.] Composition of body as per definition by Gussev [21]. Body 469–838 (617; *n* = 15) long, greatest width 100–172 (137; *n* = 15) usually between 1/3 and mid length. Haptor differentiated from body proper, 86–137 (108; *n* = 14) long, 94–149 (116; *n* = 14). Measurements of haptoral sclerites and MCO are given in Table 2. One pair of anchors of wunderi-type [21], of slightly sturdy appearance, with well-developed outer root and more prominent inner root. Outer root short with rectangular base, inner root elongated, nearly 1/2 of anchor shaft length. Shaft narrows in inner side before turning in point. Transversal bar bone-like, with rounded end. Hooks seven pairs, all of similar shape, short, fined point, well-demarcated gourd shape handle. Uneven in size, pair I and V slightly smaller. MCO composed of simple tube, narrowed into a fine tip. Accessory piece elongated, embraces tube in its half, usually lies along tube.

Remarks: The shape and size of hard parts of the specimens collected during the present study correspond with data and drawings presented by Chinabut and Lim [11]. Only the size of the marginal hooks from the present study were somewhat smaller, 16.3–23.3 μm, compared to the 23–28 μm given by Chinabut and Lim [11]. The following species, *D. pahangensis* Lim & Furtado, 1986, *D. contrarmatus* Lim & Furtado, 1984, and *D. sclerovaginalis* Lim & Furtado, 1986, are the closest congeners to *D. tapienensis*. From *D. pahangensis, D. contrarmatus* and *D. sclerovaginalis*, *D. tapienensis* differs in (1) the general morphology of anchors – the inner root is longer in *D. pahangensis*, the outer root closer to the inner root in *D. contrarmatus*, and the outer and inner root not well developed in *D. sclerovaginalis*; (2) the morphology of the hooks, and (3) the shape of a ventral bar. From other congeners of *Dactylogyrus* bearing haptoral sclerites of similar size of anchors and one transversal bar, *D. tapienensis* is similar to *D. viticulus*, but can easily be distinguished based on the size of the MCO, 83–93.4 μm for *D. tapienensis* vs. 43.5–50.7 μm for *D. viticulus*.

### *Dactylogyrus viticulus* Chinabut & Lim, 1993 (Fig. 3)

Type-host: *Barbonymus gonionotus* (Bleeker, 1850).

**Figure 3:**
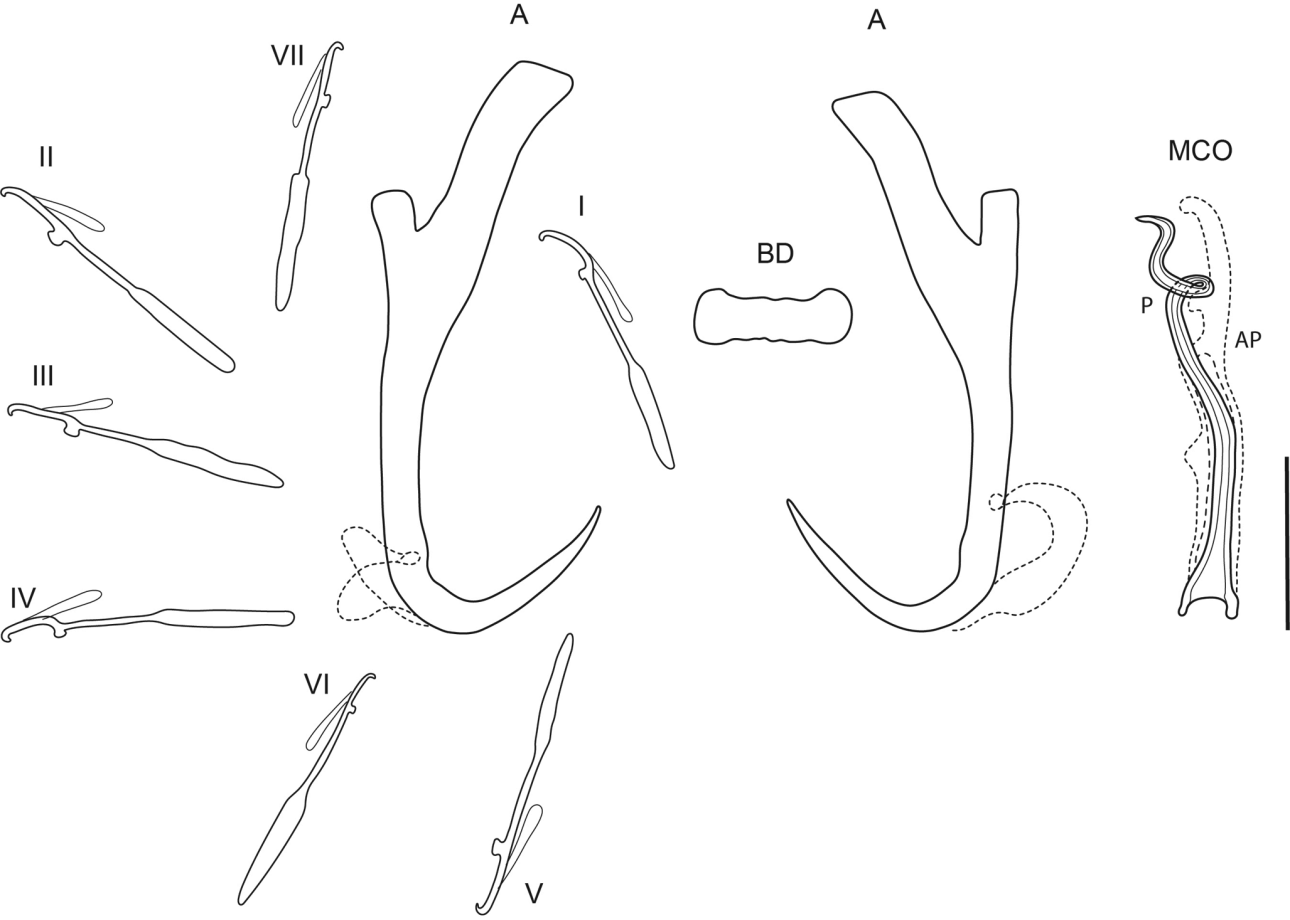
Line drawings of sclerotised structures of *Dactylogyrus viticulus* Chinabut & Lim, 1993 ex *Barbonymus schwanenfeldii.* A, anchor; BD, dorsal bar; I–VII, hooks; MCO, male copulatory organ; AP, accessory piece; P, penis. Scale bar 20 μm.

Other hosts: *Barbonymus altus* (Gunther, 1868), *Barbonymus schwanenfeldii* (Bleeker 1853).

Type-locality: Vachiralongkorn Reservoir, Kanchanaburi Province, Thailand.

Present record: Thailand.

Infection site: Gills.

Material deposited: 2 voucher specimens M-778 and 2 voucher specimens NMB P 952-3.

DNA sequence: A nucleotide sequence of partial 28S rDNA (840 bp; No. OR077125) and nucleotide sequences representing a fragment (975 bp; access. No. OR081827) including partial 18S rDNA (487 bp), and the ITS1 region (488 bp).

Redescription [based on 15 adult flattened specimens in GAP.] Composition of body as per definition by Gussev [21]. Body 446–904 (640; *n* = 13) long, greatest width 80–182 (143; *n* = 13) usually between 1/3 and midlength. Haptor differentiated from body proper, 96–150 (114; *n* = 15) long, 80–153 (112; *n* = 15). Measurements of haptoral sclerites and MCO are given in Table 2. One pair of anchors of wunderi-type [21], slightly slender appearance, with well-developed roots. Outer root short with rectangular base, inner root elongated, well 1/2 of anchor shaft length, with slightly turning end parts of roots. Shaft narrows in inner side before turning in point. Transversal bar stout shape, with slightly rounded end. Hooks seven pairs, all of similar shape, short and fine point, thin pivots and prominent handle. Hooks even in size. MCO consists of long simple tube, with single twist in distal section of tube, elongated accessory piece, rising along tube.

Remarks: The species was described by Chinabut and Lim [11] as the result of field sampling of small cyprinids and their screening for monogenean parasites. The shape of haptoral sclerites as well as the MCO morphology of the specimens collected during the present study are identical to the drawings presented in the species description [11]. In the description out of all *Dactylogyrus* spp. from small cyprinoid hosts in the area, *D. viticulus* is similar to *D. tapienensis* and *D. pahangensis.* From both species*, D viticulus* differs by having a significantly smaller MCO, 43.5–50.7 μm vs. 70–75 μm for *D. pahangensis* and 83–93.4 μm for *D. tapienensis.* However, the total length of the anchors does not differ significantly between *D. viticulus* and *D. pahangensis* (55.2–67 vs. 70–75 μm, respectively), *D. pahangensis* has a distinctively longer inner root (31–41 μm) compared to *D. viticulus* (18.8–25.3 μm).

### Phylogenetic relationships of investigated *Dactylogyrus* species

**Figure 4:**
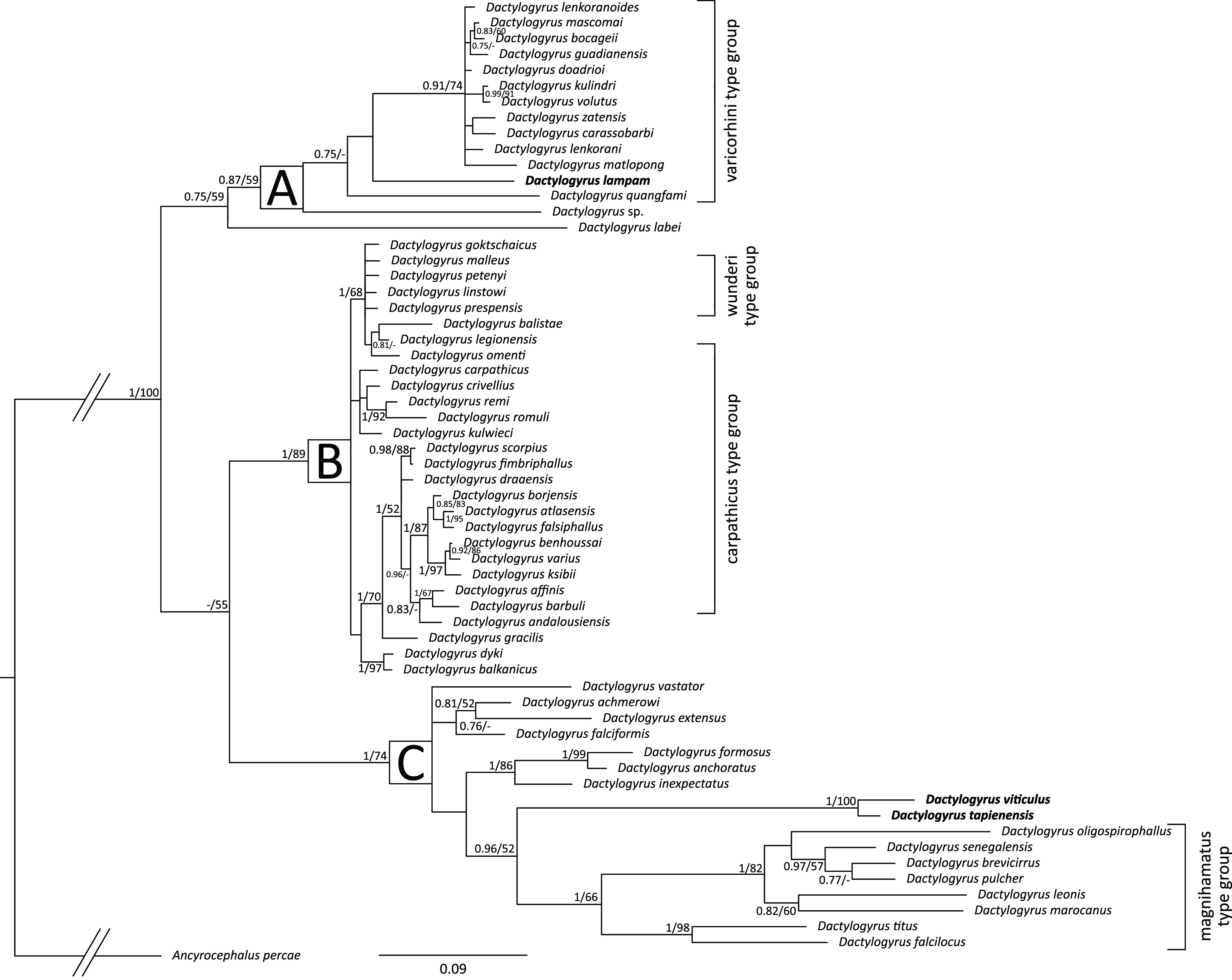
Phylogenetic tree of 60 *Dactylogyrus* spp. parasitising various cyprinid fish hosts. The tree is based on the sequences of partial genes coding 28S rRNA and rooted using *Ancyrocephalus percae*. Values at the nodes indicate posterior probabilities from BI and bootstrap values from ML analyses. Dashes indicate values below 0.75 and 50, respectively. Letters (A–C) represent specific well-supported clades. The label at the clades shows shared haptoral ventral bar morphotype for respective species. The three species from the present study are in bold.

The final sequence alignment encompassing 60 *Dactylogyrus* spp. and outgroup spanned 701 unambiguously aligned nucleotide positions. ML and BI analyses generated trees with identical topologies and BI tree with posterior probabilities and bootstrap values along respective nodes is presented in Figure 4. The phylogenetic analyses divided all the studied species into three major phylogenetic clades. The first one included all European (specifically Iberian) and African species possessing “varicorhini” morphotype of haptoral ventral bar (clade A). Within clade A were basally positioned *D. quangfami* with undescribed species *Dactylogyrus* sp. from China and *D. lampam*, and in the sister position to clade A was according to the analyses *Dactylogyrus labei* Musselieus & Gusev, 1976 from India. The second clade (clade B) included almost all other *Dactylogyrus* spp. parasitising European, North-west African and Middle Eastern cyprinids. The species of clade B possess either large “carpathicus” morphotype of ventral bar with five extremities, the triangular “rutili” morphotype, the intermediate forms with four extremities, or have a completely absent ventral bar (*Dactylogyrus balistae* Simon-Vicente, 1981 and *Dactylogyrus legionensis* Gonzales-Lanza & Alvarez-Pellitero, 1982). The last clade (clade C) included *Dactylogyrus* spp. associated with *Cyprinus carpio* L. and *Carassius* spp. which possess no ventral bar, and *Dactylogyrus* spp. parasitising large central African cyprinids (*Labeo* Cuvier) together with species parasitising African and Middle Eastern *Carassobarbus* spp. (i.e., *Dactylogyrus marocanus* El Gharbi, Birgi & Lambert, 1994 and *Dactylogyrus pulcher* Bykhovsky, 1957). The latter group (African and Middle Eastern *Dactylogyrus* spp.) is characterised by strong miniaturisation or complete absence of a connective ventral bar. The three *Dactylogyrus* spp. collected from *B. schwanenfeldii* were associated with two phylogenetically divergent *Dactylogyrus* lineages. *Dactylogyrus tapienensis* and *D. viticulus* were revealed by both analyses to be phylogenetically closely related species (uncorrected genetic distance 3.3%) and they were both in the sister position within clade C to the lineage encompassing six central-African *Dactylogyrus* spp., north-African *D. marocanus*, and Middle Eastern *D. pulcher* (uncorrected genetic distances between the species within clade C were 16.2–20.7%; for more details, see Table S1)*.* Not so well-resolved was the phylogenetic position of the *D. lampam* which was according to the current phylogenetic analyses close to the Iberian, North-African, and Middle Eastern species possessing “varicorhini” morphotype of the haptoral ventral connective bar (clade A). Based on the uncorrected *p*-distances (see Table S1), *D. lampam* is “the closest” relative to *Dactylogyrus daodrioi* El Gharbi, Renaud & Lambert, 1993, *D. lenkoranoides* El Gharbi, Renaud & Lambert, 1993, *D. zatensis* El Gharbi, Birgi & Lambert, 1994 and *D. mascomai* El Gharbi, Renaud & Lambert, 1993 with 8.9, 9.2, 9.5 and 9.5%, respectively.

## Discussion

The ornamental fish trade is a long-term, well operating industry, which every year is responsible for the relocation of a huge number of freshwater and marine fish all around the globe [10, 68]. This is associated with a risk of the introduction of ornamental fish into native environments, with many reports already confirmed worldwide, e.g., Australia [29], Canada [18], England [12] and Mexico [25]. Moreover, the ornamental fish can serve as an important pathway for the translocation of non-native parasites [14, 63, 68] as has already been confirmed in South Africa [35, 61]. The present study reporting the three species of *Dactylogyrus* on the gills of tinfoil barb imported into South Africa as ornamental fish represents another example that parasites are being moved all around the world together with their hosts, and that there is a continuously persistent risk of introduction of the non-native parasites, as previously documented [25, 30, 54].

However, *B. schwanenfeldii* can be under the natural condition parasitised by various groups of parasites, such as Trematoda [2, 37], Nematoda [23], or Myxozoa [58], but only Monogenea were found during the present survey, which included hosts bred under an artificial condition as a supply for the ornamental trade chain. It seems highly probable that monogenean species, parasites with a direct life cycle, can easily survive and live on fish under closed cultured conditions, while parasites with more complex life cycles (Nematoda, Trematoda, and Cestoda) do not complete their cycle under the closed conditions with no access to the required intermediate hosts. In the natural area of distribution of *B. schwanenfeldii*, Mekong and Chao Phraya basins, Malay Peninsula, Sumatra and Borneo [17], this fish was reported as the host for four *Dactylogyrus* spp. [11, 28], and only three of them were found on the cultured stock received from Thailand. The slightly lower parasite diversity could be explained by the loss of one of the more sensitive species during the translocation of the fish stock from its natural environment into a closed system. Such conditions can be comparable to the translocation of fish into non-native areas where parasite diversity is often lowered, defined as the enemy release hypothesis [38]. From the studies on parasites of cultured ornamental fish, it is evident that the presence of monogenean parasites is very common [60, 62, 63, 67]. As some of these parasites, such as *Dactylogyrus extensus* Mueller & Vancleave, 1932 and *Dactylogyrus vastator* Nybelin, 1924, can pose a serious concern and have been identified as a threat to indigenous fish, efforts have been made to develop non-invasive techniques for rapid and accurate identification of these species [64]. Despite newly developed techniques, screening for the presence of parasites in and on introduced/imported hosts is still mainly based on the morphometric approach for parasite identification [61, 65]. The present study supplements the original description of three *Dactylogyrus* spp. from *B. schwanenfeldii* and will undoubtedly serve as a good literature source for parasite identification.

The prevalence of *Dactylogyrus* spp. on *B. schwanenfeldii* was similar at 87% and 80% for the fish received from Sri Lanka and Thailand, respectively, and the MI was observed to be double in fish sourced from Sri Lanka (44.3) compared to Thailand (21.7). However, the study of Lim and Furtado [28] does not present a value for prevalence, but the MI can be derived from the values provided on the reports of 50 specimens per host, which is close to the observation made on fish from Sri Lanka in our study. The present study also shows that the species composition differed between the two shipments. Fish from Sri Lanka, a country that is not their natural area of distribution, were infected only by *D. lampam* (the smallest species), while fish originating in Thailand had three species. It can only be hypothesised that either the original stock has experienced loss of some more sensitive species, or the original stock already had a single species infection, as the composition of parasites can differ between studied sites [65]. Another probable explanation could be that the fishes from Sri Lanka originally had all three species, but were properly treated before being brought into a breeding facility, and the smallest parasite remained hidden between gill lamellae during the treatment bath and infection develop afterward.

Two species identified during the present study, *D. tapienensis* and *D. viticulus* bear anchors of the wunderi type, and both species have a single simple bar of the amphibotrium type [21]. They also share a similar shape of the MCO, a straight tube with an accessory piece lying along the tube of the anchoratus type. They can easily be distinguished from other congeners by the combination of shape and size of the whole complex of haptoral sclerites and MCO. The measurements of the haptoral hard parts, mainly anchors and dorsal bar, correspond well and overlap with values given by Chinabut and Lim [11]. Only the marginal hooks for *D. tapienensis* and *D. viticulus* were somewhat smaller than the size presented in the original descriptions of the species, but still overlapping (see Table 2). *Dactylogyrus lampam* is a species with haptoral sclerites of the varicorhini type, with two bars, the ventral bar having a pronounced process in the middle part, similar that of the African species *Dactylogyrus matlopong* Acosta, Truter, Malherbe, Smit, 2022. It seems that this specific detail might pose a challenge to being observed as in the drawing in Mohanta and Chandra [34], who did not show it. The sizes of haptoral sclerites of the *D. lampam* collected during the present study were slightly smaller than those given in the original species description (Table 2), except that of the marginal hook which corresponds well in its dimensions to values given by Lim and Furtado [28]. Also, Mohanta and Chandra [34] documented a slight difference in the size of sclerite between Thai and Bangladeshi specimens (see [34]).

According to the present phylogenetic analyses, three *Dactylogyrus* spp. parasitising *B. schwanenfeldii* are in a paraphyletic relationship, possibly suggesting their evolutionary divergent origin on Indo-Malaysian fish. *Dactylogyrus tapienensis* and *D. viticulus* were revealed to be phylogenetically proximal to *Dactylogyrus* spp. associated with African and Middle Eastern cyprinids possessing the magnihamatus type of haptoral ventral bar. This specific morphological element (specifically the shape of haptoral connective bars) is a phylogenetically important trait for assessing the phylogenetic relationships in *Dactylogyrus* [5, 7, 50], and thus, considering the morphological similarities, *D. tapienensis* and *D. viticulus* might appear as phylogenetically closer to *Dactylogyrus* spp. associated with *C. carpio* and *Carassius* sp. (e.g., *D. vastator, D. falciformis, D. anchoratus*), as all these species have no haptoral ventral bar. Nonetheless, the deep nodal split between the two species from *B. schwanenfeldii* and *Dactylogyrus* belonging to the magnihamatus type group suggests relatively early divergence of these two lineages; therefore, *D. tapienensis* and *D. viticulus* should represent a new phylogenetic lineage, potentially also encompassing other endemic Indonesian congeners. Similarly, *D. lampam* was in the sister position to other *Dactylogyrus* spp. possessing the same morphotype of the haptoral ventral bar, parasitising cyprinids in Africa, Europe, and the Middle East. Even though the phylogenetic relationships between lineages within clade A were not fully resolved, the topology of the phylogenetic tree and molecular differentiation also suggest early divergence of these lineages.

The phylogenetic relationships between the major cyprinid subfamilies, specifically Poropuntiinae (including *Barbonymus*), Cyprininae, and Barbinae (*sensu* [59]) are not yet fully resolved, even using a multilocus molecular approach [69]. However, considering the phylogenetic relationships of the associated *Dactylogyrus* parasites, a certain degree of cospeciation between *Dactylogyrus* and their cyprinoid hosts is expected, and we can expect that the poropuntiins will be phylogenetically closer to barbins, rather than cyprinins. Moreover, from the presence of *Dactylogyrus* spp. belonging to two such phylogenetically divergent clades on *B. schwanenfeldii*, we can hypothesize that the species of *Barbonymus* were independently colonised by *Dactylogyrus* spp. multiple times, and while *D. tapienensis* and *D. viticulus* originated from co-diversification (or intra-host speciation followed by cospeciation) with their *Barbonymus* hosts*, D. lampam* secondarily host-switched onto *Barbonymus* spp. from different cyprinoid fish in the Indonesian region. Nevertheless, in order to fully elucidate these historical diversification and dispersion processes, it would be necessary to obtain molecular data from other Indonesian *Dactylogyrus* spp. (especially for the other four species of *Barbonymus*), which are, unfortunately, still missing.

## Supplementary material

The supplementary material of this article is available at https://www.parasite-journal.org/10.1051/parasite/2023031/olm. *Table S1*. Uncorrected *p*-distances based on 28S sequences of the species included in the phylogenetic analysis.

## References

[1] Acosta AA, Truter M, Malherbe W, Smit NJ. 2022. Morphological description and molecular characterisation of *Dactylogyrus matlopong* sp. n. (Monogenea: Dactylogyridae) from the South African endemic *Labeobarbus aeneus* (Cyprinidae: Torinae). Folia Parasitologica, 69, 21. 10.14411/fp.2022.021 36227137

[2] Apiwong K, Wongsawad C, Butboonchoo P. 2018. Morphological and molecular characterization of *Haplorchoides mehrai* Pande and Shukla 1976 (Digenea: Heterophyidae) from Chiang Mai province. Helminthologia, 55, 334–342. 31662664 10.2478/helm-2018-0034 PMC6662004

[3] Benovics M, Desdevides Y, Vukić J, Šanda R, Šimková A. 2018. The phylogenetic relationships and species richness of host-specific *Dactylogyrus* parasites shaped by the biogeography of Balkan cyprinids. Scientific Reports, 8, 13006–13018. 30158640 10.1038/s41598-018-31382-w PMC6115452

[4] Benovics M, Desdevides Y, Šanda R, Vukić J, Scheifler M, Doadrio I, Sousa-Santos C, Šimková A. 2020. High diversity of fish ectoparasitic monogeneans (*Dactylogyrus*) in the Iberian Peninsula: a case of adaptive radiation? Parasitology, 147, 418–430. 31965950 10.1017/S0031182020000050 PMC10317701

[5] Benovics M, Vukić J, Šanda R, Rahmouni I, Šimková A. 2020. Disentangling the evolutionary history of peri-Mediterranean cyprinids using host-specific gill monogeneans. International Journal for Parasitology, 50(12), 969–984. 32619430 10.1016/j.ijpara.2020.05.007

[6] Benovics M, Francová K, Volta P, Dlapka V, Šimková A. 2021. Helminth communities of endemic cyprinoids of the Apennine Peninsula, with remarks on ectoparasitic monogeneans, and a description of four new *Dactylogyrus* Diesing, 1850 species. Parasitology, 148, 1003–1018. 33843503 10.1017/S0031182021000615 PMC10090784

[7] Benovics M, Nejat F, Abdoli A, Šimková A. 2021. Molecular and morphological phylogeny of host-specific *Dactylogyrus* parasites (Monogenea) sheds new light on the puzzling Middle Eastern origin of European and African lineages. Parasites & Vectors, 14, 372. 34289869 10.1186/s13071-021-04863-7 PMC8293574

[8] Benovics M, Vukić J, Šanda R, Nejat F, Charmpila EA, Buj I, Shumka S, Porcelloti S, Tarkan SA, Aksu S, Emiroğlu O, Šimková A. 2023. Monogeneans and chubs: ancient host-parasite system under the looking glass. Molecular Phylogenetics and Evolution, 179, 107667. 36400419 10.1016/j.ympev.2022.107667

[9] Bush AO, Lafferty KD, Lotz JM, Shostak AW. 1997. Parasitology meets ecology on its own terms: Margolis et al. revisited. Journal of Parasitology, 83, 575–583. 9267395

[10] Cheong L. 1996. Overview of the current international trade in ornamental fish with special reference to Singapore. In: Preventing the spread of aquatic animal diseases. (Eds. B. J. Hill and T. Hastein). Revue Scientifique et Technique, 15, 445–481. 8890375 10.20506/rst.15.2.935

[11] Chinabut L, Lim S. 1993. Seven new species of *Dactylogyrus* Diesing, 1850 (Monogenea) from *Puntius hamilton* (Cyprinidae) of Thailand. Raffles Bulletin of Zoology, 41, 47–59.

[12] Copp GH, Wesley KJ, Vilizzi L. 2005. Pathways of ornamental and aquarium fish introductions into urban ponds of Epping Forest (London, England): the human vector. Journal of Applied Ichthyology, 21, 263–274.

[13] Darriba D, Taboala GL, Doallo R, Posada D. 2012. JModelTest2: more models, new heuristics and parallel computing. Nature Methods, 9, 772. 10.1038/nmeth.2109 PMC4594756 22847109

[14] Dove AD, Ernst I. 1998. Concurrent invaders – four exotic species of Monogenea now established on exotic freshwater fishes in Australia. International Journal for Parasitology, 28, 1755–1764. 9846613 10.1016/s0020-7519(98)00134-9

[15] Ergens R. 1969. The suitability of ammonium picrate-glycerin preparing slides of lower Monogenoidea. Folia Parasitologica, 16(4), 320.

[16] Esa Y, Japning JRR, Rahim KAA, Siraj SS, Daud SK, Tan SN, Sungan S. 2012. Phylogenetic relationships among several freshwater fishes (Family: Cyprinidae) in Malaysia inferred from partial sequencing of the cytochrome b mitochondrial DNA (mtDNA). Pertanika Journal of Tropical Agricultural Science, 35(2), 307–318.

[17] Fricke R, Eschmeyer W, Van Der Laan R. 2021. Eschmeyer’s catalog of fishes. Genera, species references. http://researcharchive.calacademy.org/research/ichthyology/catalog/fishcatmain.asp.

[18] Gertzen E, Familiar O, Leung B. 2008. Quantifying invasion pathways: fish introductions from the aquarium trade. Canadian Journal of Fisheries and Aquatic Sciences, 65(7), 1265–1273.

[19] Gibson DI, Timofeeva TA, Gerasev PI. 1996. A catalogue of the nominal species of the monogenean genus *Dactylogyrus* Diesing, 1850 and their host genera. Systematic Parasitology, 35, 3–48.

[20] Guindon S, Gascuel O. 2003. A simple, fast and accurate algorithm to estimate large phylogenies by maximum likelihood. Systematic Biology, 27, 1759–1767. 10.1080/10635150390235520 14530136

[21] Gussev AV. 1985. Metazoan parasites. Part I. Key to parasites of freshwater fish of USSR, Vol. 2. Leningrad: Nauka.

[22] Ha K. 1971. New species of monogenean form freshwater fishes of Morth Vietnam. II. Parazitologia, 5, 429–440.

[23] Hafiza N, Shaharom-Harrison F. 2020. The description of Nematode in *Barbonymus schwanenfeldii* at Kenyir Lake, Terengganu. Universiti Malaysia Terengganu Journal of Undergraduate Research, 2, 31–36.

[24] Hassouna N, Michot B, Bachellerie JP. 1984. The complete nucleotide sequence of mouse 28S rRNA gene. Implications for the process of size increase of the large subunit rRNA in higher eukaryotes. Nucleic Acids Research, 12, 3563–3583. 6328426 10.1093/nar/12.8.3563 PMC318769

[25] Jiménez-García MI, Vidal-Martínez VM, López-Jiménez S. 2001. Monogeneans in introduced and native cichlids in Mexico: evidence for transfer. Journal of Parasitology, 87, 907–909. 11534657 10.1645/0022-3395(2001)087[0907:MIIANC]2.0.CO;2

[26] Kamarudin KR, Esa Y. 2009. Phylogeny and phylogeography of *Barbonymus schwanenfeldii* (Cyprinidae) from Malaysia inferred using partial cytochrome b mtDNA gene. Journal of Tropical Biology and Conservation, 5, 1–13.

[27] Katoh K, Misawa K, Kuma K, Miyata T. 2002. MAFFT: a novel method for rapid multiple sequence alignment based on Fourier transform. Nucleic Acids Research, 30, 3059–3066. 12136088 10.1093/nar/gkf436 PMC135756

[28] Lim LHS, Furtado JI. 1986. Sixteen new species of *Dactylogyrus* from Genus *Puntius* Hamilton (Cyprinidae). Folia Parasitologia, 33, 21–34.

[29] Lintermans M. 2004. Human-assisted dispersal of alien freshwater fish in Australia. New Zealand Journal of Marine and Freshwater Research, 38, 481–501.

[30] Lymbery AJ, Morine M, Kanani HG, Beatty SJ, Morgan DL. 2014. Co-invaders: the effects of alien parasites on native hosts. International Journal for Parasitology: Parasites and Wildlife, 3, 171–177. 25180161 10.1016/j.ijppaw.2014.04.002 PMC4145144

[31] Malmberg G. 1957. On a new genus of viviparous monogenetic trematode. Arkiv for Zoologi, 10, 317–329.

[32] Mendoza-Palmero CA, Blasco-Costa I, Scholz T. 2015. Molecular phylogeny of Neotropical monogeneans (Platyhelminthes: Monogenea) from catfishes (Siluriformes). Parasites & Vectors, 8, 164. 25890068 10.1186/s13071-015-0767-8 PMC4374382

[33] Mizelle JD. 1936. New species of trematodes from the gills of Illinois fishes. American Midland Naturalist, 17, 785–806.

[34] Mohanta SK, Chandra KJ. 2000. Monogenean infestations in Thai silver barb (*Barbodes gonionotus* Bleeker) and their adaptations in Bangladesh waters. Bangladesh Journal of Fisheries Research, 4(2), 147–155.

[35] Mouton A, Basson L, Impson D. 2001. Health status of ornamental freshwater fishes imported to South Africa: a pilot study. Aquarium Sciences and Conservation, 3, 313–319.

[36] Musilová N, Řehulková E, Gelnar M. 2009. Dactylogyrids (Platyhelminthes: Monogenea) from the gills of the African carp, *Labeo coubie* Rüppell (Cyprinidae), from Senegal, with descriptions of three new species of *Dactylogyrus* and the redescription of *Dactylogyrus cyclocirrus* Paperna, 1973. Zootaxa, 2241(1), 47–68.

[37] Namsanor J, Kiatsopit N, Laha T, Andrews RH, Petney TN, Sithithaworn P. 2020. Infection dynamics of *Opisthorchis viverrine* Metacercariae in Cyprinid fishes from two endemic areas in Thailand and Lao PDR. American Journal of Tropical Medicine and Hygiene, 102, 110–116. 31701859 10.4269/ajtmh.19-0432 PMC6947784

[38] Pettersen RA, Ostbye K, Holmen J, Vollestad LA, Mo TA. 2016. *Gyrodactylus* spp. Diversity in native and introduced minnow (*Phoxinus phoxinus*) populations: no support for “the enemy release” hypothesis. Parasites & Vectors, 9, 51. 26822543 10.1186/s13071-016-1306-y PMC4730603

[39] Pugachev ON, Gerasev PI, Gussev AV, Ergens R, Khotenowsky I. 2009. Guide to Monogenoidea of freshwater fish of Palaearctic and Amur regions. Milan: Ledizione-LediPublishing, 568 p.

[40] Rahmouni I, Řehulková E, Pariselle A, Rkhami OB, Šimková A. 2017. Four new species of *Dactylogyrus* (Monogenea: Dactylogyridae) parasitizing the gills of northern Moroccan *Luciobarbus* (Cyprinidae): morphological and molecular characterization. Systematic Parasitology, 94, 575–591. 28432566 10.1007/s11230-017-9726-4

[41] Rambaut A, Drummond AJ, Xie D, Baele G, Suchard MA. 2018. Posterior summarization in Bayesian phylogenetics using Tracer 1.7. Systematic Biology, 67, 901–904. 29718447 10.1093/sysbio/syy032 PMC6101584

[42] Raphahlelo ME, Přikrylová I, Matla MM. 2020. *Dactylogyrus* spp. (Monogenea, Dactylogyridae) from the gills of *Enteromius* spp. (Cypriniformes, Cyprinidae) from the Limpopo Province, South Africa with descriptions of three new species. Acta Parasitologica, 65, 396–412. 32056086 10.2478/s11686-020-00175-5

[43] Řehulková E, Seifertová M, Přikrylová I, Francová K. 2018. Monogenea, in A guide to the parasites of African freshwater fishes, Scholz T, Vanhove MPM, Smit N, Jayasundera Z, Gelnar M, Editors. RBINS′ Scientific Publication Unit, ABC Taxa: Brussels. p. 185–243.

[44] Řehulková E, Benovics M, Šimková A. 2020. Uncovering the diversity of monogeneans (Platyhelminthes) on endemic cypriniform fishes of the Balkan Peninsula: new species of *Dactylogyrus* and comments on their phylogeny and host-parasite associations in a biogeographic context. Parasite, 27, 66. 33231549 10.1051/parasite/2020059 PMC7685236

[45] Řehulková E, Rahmouni I, Pariselle A, Šimková A. 2021. Integrating morphological and molecular approaches for characterizing four species of *Dactylogyrus* (Monogenea: Dactylogyridae) from Moroccan cyprinids, with comments on their host specificity and phylogenetic relationships. PeerJ, 9, 1067. 10.7717/peerj.10867 PMC8000462 33828906

[46] Ronquist F, Teslenko M, van der Mark P, Ayres DL, Darling A, Höhna S, Larget B, Liu L, Suchard MA, Huelsenbeck JP. 2012. MrBayes 3.2: efficient Bayesian phylogenetic inference and model choice across large model space. Systematic Biology, 61, 539–542. 22357727 10.1093/sysbio/sys029 PMC3329765

[47] Šimková A, Plaisance L, Matějusová I, Morand S, Verneau O. 2003. Phylogenetic relationships of the Dactylogyridae Bychowsky, 1933 (Monogenea: Dactylogyridae): the need for the systematic revision of the Ancyrophalinae Bychowsky, 1937. Systematic Parasitology, 54, 1–11. 12567005 10.1023/a:1022133608662

[48] Šimková A, Morand S, Jobet E, Gelnar M, Verneau O. 2004. Molecular phylogeny of congeneric monogenean parasites (*Dactylogyrus*): a case of intrahost speciation. Evolution, 58, 1001–1018. 15212381 10.1111/j.0014-3820.2004.tb00434.x

[49] Šimková A, Matějusová I, Cunningham CO. 2006a. A molecular phylogeny of the Dactylogyridae sensu Kritsky & Boeger (1989) (Monogenea) based on the D1–D3 domains of large subunit rDNA. Parasitology, 133, 43–53. 16515727 10.1017/S0031182006009942

[50] Šimková A, Verneau O, Gelnar M, Morand S. 2006b. Specificity and specialization of congeneric monogeneans parasitizing cyprinid fish. Evolution, 60, 1023–1037. 16817542

[51] Šimková A, Pečínková M, Řehulková E, Vyskočilová M, Ondračková M. 2007. *Dactylogyrus* species parasitizing European *Barbus* species: morphometric and molecular variability. Parasitology, 134, 1751–1765. 17662164 10.1017/S0031182007003265

[52] Šimková A, Benovics M, Rahmouni I, Vukić J. 2017. Host-specific Dactylogyrus parasites revealing new insights on the historical biogeography of Northwest African and Iberian cyprinid fish. Parasites & Vectors, 10, 58. 29183392 10.1186/s13071-017-2521-x PMC5706372

[53] Šimková A, Řehulková E, Choudhury A, Seifertová M. 2022. Host-specific parasites reveal the history and biogeographical contacts of their hosts: the monogenea of Nearctic cyprinoid fishes. Biology, 11(2), 223. 35205096 10.3390/biology11020229 PMC8869197

[54] Smit NJ, Malherbe W, Hadfield KA. 2017. Alien freshwater fish parasites from South Africa: diversity, distribution, status and the way forward. International Journal for Parasitology: Parasites and Wildlife, 6, 386–401. 30951573 10.1016/j.ijppaw.2017.06.001 PMC5715218

[55] Stamatakis A. 2006. RAxML-VI-HPC: maximum likelihood-based phylogenetic analyses with thousands of taxa and mixed models. Bioinformatics, 22, 2688–2690. 16928733 10.1093/bioinformatics/btl446

[56] Stamatakis A. 2014. RAxML version 8: a tool for phylogenetic analyses and post-analysis of large phylogenies. Bioinformatics, 30, 1312–1313. 24451623 10.1093/bioinformatics/btu033 PMC3998144

[57] Stout CC, Tan M, Lemmon AR, Lemmon EM, Armbruster JW. 2016. Resolving Cypriniformes relationships using an anchored enrichment approach. BMC Evolutionary Biology, 16, 1–13. 27829363 10.1186/s12862-016-0819-5 PMC5103605

[58] Székely Cs, Shaharom-Harrison F, Cech G, Ostoros G, Molnár K. 2009. Myxozoan infections in fishes of the Tasik Kenyir water reservoir, Terengganu, Malaysia. Diseases of Aquatic Organisms, 83, 37–48. 19301635 10.3354/dao01991

[59] Tan M, Armbruster JW. 2018. Phylogenetic classification of extant genera of fishes of the order Cypriniformes (Teleostei: Ostariophysi). Zootaxa, 4476, 006–039. 10.11646/zootaxa.4476.1.4 30313339

[60] Tancredo KR, Martins ML. 2019. Three previous recorded species of *Dactylogyrus* Diesing, 1850 (Monogenea: Dactylogyridae) infecting cultured *Carassius auratus* in southern Brazil. Journal of Parasitic Diseases, 43(3), 522–527. 31406420 10.1007/s12639-019-01121-7 PMC6667508

[61] Tavakol S, Halajian A, Smit WJ, Hoffman A, Luus-Powell WJ. 2017. Guppies (*Poecilia reticulata*) introducing an alien parasite, *Camallanus cotti* (Nematoda: Camallanidae) to Africa, the first report. Parasitology Research, 116(12), 3441–3445. 29063195 10.1007/s00436-017-5657-x

[62] Thilakaratne I, Rajapaksha G, Hewakopara A, Rajapakse R, Faizal A. 2003. Parasitic infections in freshwater ornamental fish in Sri Lanka. Diseases of Aquatic Organisms, 54, 157–162. 12747641 10.3354/dao054157

[63] Trujillo-González A, Becker JA, Hutson KS. 2018. Parasite dispersal from the ornamental goldfish trade. Advances in Parasitology, 100, 239–281. 29753340 10.1016/bs.apar.2018.03.001

[64] Trujillo-González A, Edmunds RC, Becker JA, Hutson KS. 2019. Parasite detection in the ornamental fish trade using environmental DNA. Scientific Reports, 9, 5173. 30914693 10.1038/s41598-019-41517-2 PMC6435732

[65] Truter M, Přikrylová I, Weyl OL, Smit NJ. 2017. Co-introduction of ancyrocephalid monogeneans on their invasive host, the largemouth bass, *Micropterus salmoides* (Lacepéde, 1802) in South Africa. International Journal for Parasitology: Parasites and Wildlife, 6, 420–429. 30951569 10.1016/j.ijppaw.2017.06.002 PMC5715217

[66] Truter M, Smit NJ, Malherbe W, Přikrylová I. 2021. Description of *Gyrodactylus paludinosus* sp. nov. (Monogenea: Gyrodactylidae) from the Straightfin Barb, *Enteromius paludinosus* (Peters, 1852), in South Africa. Acta Parasitologica, 67(1), 446–453. 34677799 10.1007/s11686-021-00480-7

[67] Tu X, Ling F, Huang A, Wang G. 2015. An infection of *Gyrodactylus kobayashii* Hukuda, 1940 (Monogenea) associated with the mortality of goldfish (*Carassius auratus*) from central China. Parasitology Research, 114(2), 737–745. 25471903 10.1007/s00436-014-4241-x

[68] Whittington RJJ, Chong R. 2007. Global trade in ornamental fish from an Australian perspective: the case for revised import risk analysis and management strategies. Preventive Veterinary Medicine, 81, 92–116. 17485126 10.1016/j.prevetmed.2007.04.007

[69] Yang L, Sado T, Hirt VM, Pasco-Viel E, Arunachalm M, Li J, Wang X, Freyhof J, Saitoh K, Simons AM, Miya M, He S, Mayden RL. 2015. Phylogeny and polyploidy: resolving the classification of cyprinine fishes (Teleostei: Cypriniformes). Molecular Phylogenetics and Evolution, 85, 97–116. 25698355 10.1016/j.ympev.2015.01.014

[70] Zakaria-Ismail M. 1990. Cyprinid fishes of the Genus *Cyclocheilichtys* in Peninsular Malaysia. Malayan Nature Journal, 44, 109–121.

